# Optimizing the Health Management Information System in Uttar Pradesh, India: Implementation Insights and Key Learnings

**DOI:** 10.9745/GHSP-D-21-00632

**Published:** 2022-08-30

**Authors:** Ankita Meghani, Anand B. Tripathi, Huzaifa Bilal, Shivam Gupta, Ravi Prakash, Vasanthakumar Namasivayam, James Blanchard, Shajy Isac, Pankaj Kumar, B.M. Ramesh

**Affiliations:** a Johns Hopkins Bloomberg School of Public Health, Department of International Health, Baltimore, MD, USA.; b India Health Action Trust, Lucknow, India.; c Institute for Global Public Health, Department of Community Health Sciences, University of Manitoba, Winnipeg, Canada.; d National Health Mission, Government of Uttar Pradesh, Lucknow, India.

## Abstract

An effective health management information system (HMIS) that captures accurate, consistent, and relevant data in a timely fashion can enable better planning and monitoring of health programs and improved service delivery, in turn helping increase the impact of different interventions. In 2009, the Government of Uttar Pradesh (GOUP) implemented HMIS, India’s national-level health information platform. However, key challenges, including difficulties in accessing the data through a web-based portal and its limited relevance to decision making and managerial needs, reduced its usability at the district and state levels. In 2015, with the support of the Uttar Pradesh Technical Support Unit, the GOUP created its own data platform, the Uttar Pradesh HMIS (UP-HMIS), to capture data elements missing from HMIS but important to UP decision makers. The UP-HMIS was redesigned to capture these data elements to holistically measure and monitor the performance of health programs and inform decision making at the district and state levels. In addition, the GOUP implemented complementary initiatives to improve data quality and data use processes. To improve HMIS data quality, the GOUP established data validation committee meetings at the block, district, and state levels. To promote the use of these validated data, in 2017, the GOUP developed and implemented the UP Health Dashboard, which ranks each of UP’s 75 districts on a set of key HMIS priority health indicators. These policy guidelines have brought greater attention to UP-HMIS data quality and use; however, additional strengthening is required to improve the quality and use of HMIS data. There is a need to increase the overall capacity and understanding of HMIS data, not only for staff with specific data-related responsibilities but also for program managers and senior decision makers.

## Abstract (precis)

The Uttar Pradesh Health Management Information System has allowed managers across all levels of the state’s health system to access routinely collected data through a comprehensive online portal, contributing to a culture of information use.

## Teaser

**Key Findings:**

- The Uttar Pradesh Health Management Information System (UP-HMIS) has allowed managers across all levels of the health system to access routinely collected data through an online portal.
- The UP-HMIS built on the national HMIS by capturing appropriate data elements; furthermore, it integrated the collection of inputs and processes indicators that had been collected via 80 paper-based forms that ran in parallel with the national HMIS.
- To strengthen UP-HMIS implementation and create a culture of high-quality data use, trainings on improving data quality (e.g., through the implementation of regular audits and supportive supervision) and data use (e.g., through monthly review meetings) were implemented across all levels of the health system.

**Key Implication:**

- Authorities should encourage continued data use and performance improvement by investing in training for local-level staff who analyze UP-HMIS data for decision making.

## INTRODUCTION

Routine health information system or health management information system (HMIS) data enables effective decision making by supporting multiple critical health system functions; including enhancing client and individual care; improving the management of health units and facilities; and supporting the policy, planning, and coordination of health programs.^1^

An effective HMIS captures accurate, consistent, and relevant data in a timely fashion. This facilitates informed planning and monitoring and improved service delivery, increasing the impact of different health interventions and programs. Particularly in Uttar Pradesh, India, which continues to have one of the country’s highest infant, maternal, and under-5 child mortality rates, strengthening the existing health systems, including the HMIS, has been viewed as a key priority.^2^^,^^3^

The Government of India (GOI) has prioritized implementing district-level electronic routine health information databases and integrating existing health information systems.^4^ The GOI has identified HMIS as a priority because periodic surveys, such as the National Family Health Surveys^5^ (done every 5 years), do not capture the relevant health data required for routine planning and monitoring. Before 2008, the GOI collected specific state-level health data using a paper-based system; however, this was insufficient for meeting planning and monitoring needs. To address this challenge, in 2005, the GOI launched the National Rural Health Mission—now known as the National Health Mission—to improve the health care of rural populations, especially poor and marginalized groups.^6^ Part of the initiative included establishing systems like the HMIS to capture information on health services performance at the lowest levels of the health system (such as the facility, block, and district levels) for regular tracking and planning. In 2008, the GOI launched the HMIS portal, leading to a faster flow of information on specified data elements from the facility level up to the block, district, state, and national levels.

The national HMIS is a web-based monitoring information system that has been implemented and adopted across all states. Through the HMIS, the GOI monitors a number of national health schemes, tracks the performance of district health systems, and captures data on (1) service delivery, such as number and types of facility-, block-, and district-level health services delivered; (2) training, such as numbers of medical and paramedical staff trained; and (3) infrastructure, such as the number of available human resources, equipment, specialty, and other services provided by a facility.^7^ All states and union territories submit HMIS data every month. In 2019, the GOI introduced a new web portal with improved performance and functionality that was first launched in UP and 4 other states^8^ and now has been implemented nationwide.

The Government of Uttar Pradesh (GOUP) first implemented the national HMIS platform in 2009. However, several challenges affected both data quality and usability for programmatic decision making in Uttar Pradesh. These included (1) difficulty in downloading the data from the national portal for more detailed analyses by the state to identify gaps in data availability and quality, as well as programmatic gaps; (2) limited number of data fields in the national HMIS, which led the state to design and implement a separate manual (paper-based) data collection system to capture missing indicators; and (3) challenges with integrating other state health data systems, such as the Human Resource Management System and the Drugs and Vaccines Distribution Management System, with the national HMIS.

To understand these issues, in 2013, the GOUP, in collaboration with the UP-TSU, conducted a study that revealed redundancies in data forms and data elements being collected, and the absence of crucial elements necessary for effective planning and programming at the block and district levels (Box).^9^^–^^11^ Although subcenters, primary health centers, community health centers, and district hospitals all reported data to the national HMIS, the data were not consistently used to inform management and programmatic decision making at the facility, district, and state levels. HMIS data elements were viewed as less relevant for day-to-day decisions because the format lacked data on input and process indicators, which program managers found more informative. Therefore, many program managers across the block, district, and state levels collected these data through separate paper-based or manual data reports parallel to the HMIS.

BOX **Establishment of the Uttar Pradesh Technical Support Unit**

In October 2013, the University of Manitoba and the India Health Action Trust established the Uttar Pradesh Technical Support Unit (UP-TSU) as a part of a memorandum of understanding between the Government of Uttar Pradesh (GOUP) and the Bill & Melinda Gates Foundation to support the GOUP in enhancing the efficiency and effectiveness of implementing the reproductive, maternal, neonatal, child, and adolescent health program. One of the UP-TSU’s key activities was to support the GOUP in improving the collection, quality, and use of routinely collected health data across all levels of the health system in the HMIS, as well as data reported in national-level initiatives, such as the Mother and Child Tracking System (MCTS), which is separate from the national HMIS. Specifically, the MCTS captures individual beneficiary-level data on the full range of health and immunizations services for pregnant women and children up to 5 years of age,^11^ unlike the HMIS, which captures facility-level services that are aggregated from registers. The MCTS was specifically developed to meet the data needs of health workers that were unmet by the HMIS because it captures a due list of antenatal and child immunization services that need to be provided by catchment area.^12^

When reflecting on the existing national data systems and platforms, such as the HMIS and Mother and Child Tracking System, the GOUP recognized the need for its own system to meet its health data requirements. In 2015, with the support of the Uttar Pradesh Technical Support Unit (UP-TSU), the GOUP began developing its own data platform known as the Uttar Pradesh HMIS (UP-HMIS), which was rolled out in 2017.^12^ The 3 primary objectives for UP-HMIS were to (1) capture the data absent from the national HMIS that UP program managers required for decision making, (2) provide decision makers at different levels of the health system with relevant data to holistically measure the performance of health programs, and (3) integrate various government data portals and manual or paper-based reports into a centralized electronic data portal. Specifically, the GOUP saw the development of UP-HMIS as a first step toward centralizing all the health data in the state, and it became a part of a broader vision for a comprehensive and integrated digital government health data portal. When the GOI updated the HMIS portal in 2019, the new web portal was implemented across the state of UP and the changes were incorporated into the UP-HMIS.

The Government of Uttar Pradesh saw the development of the UP-HMIS as a first step towards centralizing all the health data in the state, and it became a part of a broader vision for a comprehensive and integrated digital government health data portal.

We describe the strategies taken by the GOUP in collaboration with the UP-TSU to design, implement, and update the UP-HMIS and summarize the key strengths, limitations, and lessons learned from the process. We conclude with a discussion of further plans to improve the UP-HMIS.

## LANDSCAPE ANALYSIS: UNCOVERING THE BARRIERS AFFECTING HMIS IMPLEMENTATION

Before the policy decision to develop its own UP-HMIS, the GOUP, with support from the UP-TSU, initiated analyses to systematically understand the ground realities and barriers affecting HMIS implementation (Table 1). By early 2014, the UP-TSU had recruited and trained staff to support existing national HMIS data collection and analyses, as well as support the broader data efforts for the landscape analysis to uncover the challenges to national HMIS implementation. Staff included 25 district monitoring and evaluation (M&E) specialists, who were placed in 25 high-priority districts identified by the GOI and the GOUP, as well as 100 nurse mentors and 100 block community supervisors located in 100 of the 294 focus blocks within the 25 high-priority districts.

**TABLE 1.** Barriers Affecting the Availability, Quality, and Use of India National HMIS Administrative Data, and Subsequent Activities and Policies Addressing the Barriers,^a^ 2020

	**Barriers Affecting Existing Availability, Use, and Quality of National HMIS Data**	**Major Activities Implemented During Landscape Analysis to Understand and Address Barriers**	**GOUP Policy Actions Taken to Address Barriers**
Data availability	Untimely or no reporting of data from both public and private facilities; many were absent from HMIS Duplication of data and reporting across different manual paper-based forms (several data elements included in existing routine data sources were considered irrelevant or duplicative) Data elements that program managers found useful in daily decision making were not listed in the existing national HMIS or paper-based reports	Mapped all public and private health facilities in UP Reviewed and reduced duplication of data elements across 80 reporting forms Revised reporting forms to include relevant data for decision making Developed a definition guide for each data element Conducted auxiliary nurse midwives orientation workshops to increase familiarity with forms and data elements Established state data portal allowing easy data download and analysis	Outlined reporting timelines for all the health facilities Required private health facilities to report data to UP-HMIS Required updated UP-HMIS formats to be printed and made available to all facilities
Data quality	Processes for data quality review were absent, poorly implemented, and inadequate A routine platform to review the quality of routine data missing from block, district, and state levels Manual paper-based data collection reduced the timeliness, completeness, and accuracy of reporting	Conducted training sessions for data-related managers at the block and district levels on how to implement data validation checks	Required the establishment of data validation committee meetings at the block, district, and state levels Replaced manual paper-based reporting with digital UP-HMIS reporting across all districts
Data use	Difficulty downloading data from the national HMIS to conduct analysis No uniform framework for review meeting and data use Review mostly focused on logistics issues rather than using data to address utilization/coverage issues Complexity of data made analysis difficult Lack of human resources (both in number and skill) to analyze the data for use	Developed UP Health Dashboard and shared login with all block, district, and state level program managers Shared monthly district-level rankings with districts Disseminated guidelines, including a framework on how to use data to address program barriers, develop action plans, and conduct review meetings	Directed all programs to review data based on UP-HMIS and the UP Health Dashboard to promote a culture of data-informed decision making

Abbreviations: GOUP, Government of Uttar Pradesh; HMIS, health management information system; UP, Uttar Pradesh; UP-HMIS, Uttar Pradesh Health Management Information System.

^a^Source: Authors’ analysis.

In September 2014, the UP-TSU conducted a landscape analysis consisting of (1) supportive supervision by the district M&E specialists, nurse mentors, and block community supervisors across 100 blocks in 25 high-priority districts, which relied on using a standardized tool consisting of open- and close-ended questions, to understand the implementation of existing national HMIS and data management processes at the block and district levels; (2) analyses of existing HMIS data to assess their reliability and validity; (3) observations of program review meetings at the district and state levels; and (4) the document review of guidelines published by the government (e.g., program implementation plans, district health society meeting guidelines, and HMIS reporting guidelines) and reports (e.g., program/expenditure reports) submitted by the blocks and districts. Findings from this landscape analysis revealed technical, process-related, and organizational challenges that hindered HMIS implementation and subsequently affected data quality and use.

A landscape analysis revealed technical, process-related, and organizational challenges that hindered HMIS implementation and subsequently affected data quality and use.

### Technical Challenges

Duplicative and sometimes discrepant data were being collected between the national HMIS and the manual paper-based program reports, which were identified by comparing which data elements included in manual paper-based reports were missing from the HMIS. Furthermore, supportive supervision visits revealed that each program area had a separate data collection format, which had been developed to compensate for the various data elements that were required to monitor several national- and state-level health schemes but that were missing from the HMIS. However, most of these forms and formats were not synchronized and several duplications in forms and data elements were observed. Important data elements required for planning and monitoring health programs were also missing from paper-based reports. These findings highlighted data gaps at the block, district, and state levels, and the reliance on paper-based reports also pointed to a larger need to update the national HMIS. In its absence, identifying the data elements on manual paper-based program reports that were not reported in HMIS became a priority, especially as state-level program directors and managers relied on indicators included in the manual program reports to make decisions about program performance.

Another technical challenge in the national HMIS was the lack of easy access to raw data, making regular analysis more difficult. The national HMIS required that monthly facility data be entered into a separate portal, but the portal lacked provisions for program managers at the state, district, or block levels to easily download it for review and analysis. For instance, creating a monthly HMIS data file for the state required separately downloading the dataset for each health facility in the state, then compiling them—a process that could take at least 2 weeks.

Technical challenges were also noted at an individual level, as many block- and district-level program managers had limited capacity to interpret and analyze data elements in HMIS. For example, they tended to have an inadequate understanding of the types of data elements being collected, as well as how to analyze and use them to plan, manage, and target health programs.

The availability of relevant data formats across different levels of the health system also affected HMIS quality. Across the health facilities surveyed in 100 blocks, only 48% had the correct formats available based on supportive supervision visits conducted.^13^ Other factors contributing to incomplete reporting included the absence of multiple public and private facilities from the HMIS and insufficient reporting by private-sector facilities (Supplement 1).^13^

### Process-Related Challenges

The landscape analysis also uncovered challenges resulting from poor processes affecting the timeliness and completeness of reporting across the surveyed blocks of high-priority districts. Although the expected monthly data collection window was the 21st–20th (21st of the previous month to the 20th of the current month), only 49% of facilities reported on time.^13^ Reporting periods tended to differ by district, often based on the convenience of when the district magistrate held a district health society meeting. Consequently, data would often be aggregated in advance of the meeting, not necessarily to meet the outlined manual program report guidelines. This resulted in districts forwarding data to the state at different intervals according to their own monthly reporting periods (e.g., 15th–16th, 26th–25th, 1st–30th) that diverged from the state guidelines. An analysis of the existing national HMIS also revealed that districts had inadequate mechanisms in place to validate the data. Often data uploaded to the HMIS were blank and inaccurate.

The landscape analysis uncovered challenges resulting from poor processes affecting the timeliness and completeness of reporting across the surveyed blocks and districts.

More broadly, the landscape analysis revealed an inconsistency in how data sources were being used by the GOI and the GOUP to monitor program progress. Although the GOI only used the HMIS data for program review—specifically, to grade health facilities and establish a program implementation plan—the GOUP intended to primarily use the manual reports to make program review decisions and to track district performance.

However, processes for data review were also found to be weak. While the executive committee meeting platform (monthly meetings chaired by the district’s chief medical officer and attended by block-level health staff) existed across the districts, there were no clear guidelines or agendas that supported a data-informed approach to decision making during these meetings. Meetings tended to focus on resolving logistical issues rather than strategically thinking about how best to review the data to identify and address gaps in service utilization and coverage. Additionally, districts noted a lack of understanding about data sources and portals to review during these meetings, and how to then analyze and apply the data for program decision making.

### Organizational Challenges

The landscape analysis also revealed broader organizational gaps affecting HMIS performance and a lack of culture of data quality and use. For example, there were no policies and guidelines on HMIS reporting processes across different levels of the health system. In the absence of organizational guidance or trainings, existing data quality review and use mechanisms were inadequately implemented at the block, district, and state levels. Many block- and district-level staff responsible for managing and running health programs needed hands-on training on how to analyze data, check data quality, and use those data to guide programmatic decision making. Similarly, supportive supervision visits for data quality and use were weakly implemented, if at all, and there were no structured guidelines or checklists to support their implementation.

## DESIGNING AND IMPLEMENTING INTERVENTIONS TO ADDRESS THE NATIONAL HMIS CHALLENGES

**FIGURE 1:**
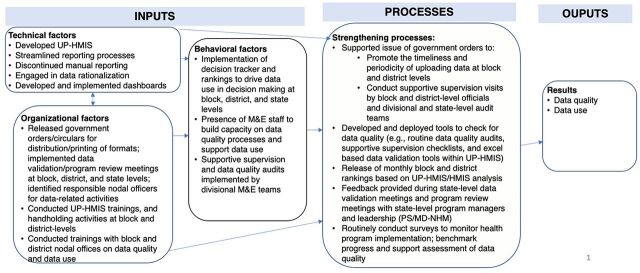
Mapping of Activities Conducted by UP-TSU, in Collaboration With the GOUP to Strengthen the Inputs and Processes to Enhance Overall Quality and Use of HMIS/UP-HMIS Data in Uttar Pradesh Abbreviations: HMIS, health management information system; M&E, monitoring and evaluation; PS/MD-NHM, Principal Secretary/Mission Director- National Health Mission; UP-HMIS, Uttar Pradesh Health Management Information System.

The Performance of Routine Immunization System Management (PRISM) framework is useful for evaluating HMIS performance in any health system and identifying key areas for strengthening and integration.^14^ Although the PRISM framework was not explicitly used to design the UP-HMIS and related interventions to strengthen the system, we use it in this article to describe the interventions that the GOUP and UP-TSU developed to address the identified gaps, including seeking technical and organizational-level inputs and developing key processes to improve the data collection, quality, and use of HMIS/UP-HMIS data (Figure 1).

### Addressing the Technical Challenges Through the UP-HMIS Web Portal

The UP-HMIS was developed primarily to address the technical limitations of the national HMIS and address UP’s data needs to improve program monitoring and planning. To develop the UP-HMIS, a comprehensive review of all available reporting formats was conducted by the UP-TSU in consultation with the GOUP program heads to ensure that captured data were unique and relevant for decision-making and management needs. Multiple consultation workshops were held from January–February 2017 with directors and joint directors from the Department of Medical Health and Family Welfare, general managers from the National Health Mission, and other partners to assess the relevance and use of data for decision making at the facility, block, district, and state levels and reduce duplication across different reporting formats. To streamline paper-based reporting, the GOUP implemented a policy to create paperless reporting and review through the UP-HMIS. Based on this decision, the UP-TSU also began mapping all the paper-based formats used for GOUP and other allied government partners in the state, initially identifying 80 formats. After reviewing these data formats, 32 duplicate formats were removed, and the 48 remaining unique formats were used to develop the UP-HMIS implemented in 2017.

To streamline paper-based reporting, the GOUP implemented a policy to create paperless reporting and review through the UP-HMIS.

The UP-HMIS was developed using District Health Information System-2 (DHIS-2), an open-source, web-based HMIS platform that has been implemented in 67 low- and middle-income countries. Using DHIS-2 made it possible to analyze UP-HMIS data in real time, generate data visualizations, and create a health data warehouse. Furthermore, the UP-TSU also built in automated data quality and validation checks to help managers at different levels of the health system proactively identify and monitor data entry errors. The automatic locking of the data in UP-HMIS also helped harmonize data reporting periods and enabled consistent examination of data quality indicators.

In addition to prioritizing the relevance of collected data, the GOUP and UP-TSU also focused on improving the number of public and private health facilities reporting to the UP-HMIS, as well as the completeness of data in each facility report. In 2014–2015, the UP-TSU mapped and verified the listings of public and private health facilities in 25 high-priority districts, including the updating and removal of facilities that no longer existed or were mislabeled. The list of facilities from different data sources (HMIS, Mother and Child Tracking System) were mapped and developed into a unique list and later verified by field visits. Overall, this activity resulted in the integration of 4,143 additional health facilities into the UP-HMIS (Supplement 1).

The UP-HMIS was designed such that data were locked within the system at the block level by the 15^th^ of every month and included automated validation checks to signal data errors (useful to examine data outliers). This technical design also facilitated data quality checks at the block level and district levels to be conducted in advance of these meetings (as explained in later sections).

A review of UP-HMIS data quality from the time it was first implemented in 2017 to 2019 revealed that several data elements were not being used by program officers for decision making (Table 2). This prompted a UP-HMIS data rationalization activity in June–September 2019. At the time, the GOUP also decided to integrate into UP-HMIS relevant process-level data from other portals, including the Human Resources Management Systems, the Drugs and Vaccine Distribution Management System, and the Reproductive and Child Health Portal (previously known as the Mother and Child Tracking System). This activity helped streamline data collection and lays the groundwork for future data rationalization processes.

**TABLE 2.** Reduction in the Number of Data Elements Collected in India National HMIS and UP-HMIS Formats^a^ by Facility Types, 2014–2019

		**HMIS Data Elements**		**UP-HMIS Data Elements**	
Section	Health Domains and Subdomains	2014	2017^b^	2017	2019^c^
A	Human resources	0	0	102	0
B	Training	1	0	39	16
C	Availability of RMNCH+A^d^ drugs, supplies, and equipment as per 5X5 matrix	0	27	113	32
D	Performance indicator				
D.1	Hospital and laboratory services	40	75	19	26
D.2	Maternal/newborn health	42	54	59	43
D.3	Maternal complication	5	2	84	48
D.4	Newborn complication	0	1	36	24
D.5	Child immunization	38	51	0	0
D.5	Child health	9	14	29	12
D.6	Family planning	18	25	28	0
D.7	Adolescent and reproductive health	0	6	0^c^	0
D.8	JSSK^e^ and grievance redressal	0	0	16	16
D.9	NVBDCP and RNTCP	3	15	0	0
E	Death details	Line listing	41	0	0
F	Process indicator and ASHA grievance redressal	0	0	43	5
G	Home-based newborn care	0	0	19	19
H	Village health and nutrition days/community process	0	0	21	6
I	National program (blindness)	6	0	0	0
J	Other (Janani Suraksha Yojana and urban health)	0	0	0	3
	Total	162	311	608	250

Abbreviations: ASHA, accredited social health activist; GOUP, Government of Uttar Pradesh; HMIS, health management information system; NVBDCP, National Vector Borne Disease Control Programme; RMNCH+A, reproductive, maternal, neonatal, child, and adolescent health; RNTCP, Revised National TB Control Programme; UP, Uttar Pradesh; UP-HMIS, Uttar Pradesh Health Management Information System; UP-TSU, Uttar Pradesh Technical Support Unit.

^a^Source: UP-TSU analyses of HMIS and UP-HMIS.

^b^In 2017, the Government of India updated the national HMIS facility-wise formats as per feedback received from the states.

^c^A data rationalization activity of UP-HMIS elements was conducted during June–September 2019 to remove several elements not used for decision making and to add new programs at the state and national levels. In UP-HMIS, there are rows with zero data elements because these data are captured in HMIS and then integrated in UPHMIS portal for data review. The UP-HMIS numbers are additional data elements beyond what is found in the HMIS.

^d^RMNCH+A standards.

^e^Janani Shishu Suraksha Karyakaram (JSSK) covers the delivery costs, including for cesarean delivery, for all pregnant women delivering in public health facilities.

### Addressing Process and Organizational Challenges Through GOUP Policies

An enabling policy environment, technical assistance to enhance district-level capacities for data quality and use, and the implementation of several initiatives that strengthened the processes for data quality and use at the block, district, and state levels were critical for addressing the organizational and process-related barriers that affected UP-HMIS performance.

#### Establishing an Enabling Policy Environment

**FIGURE 2:**
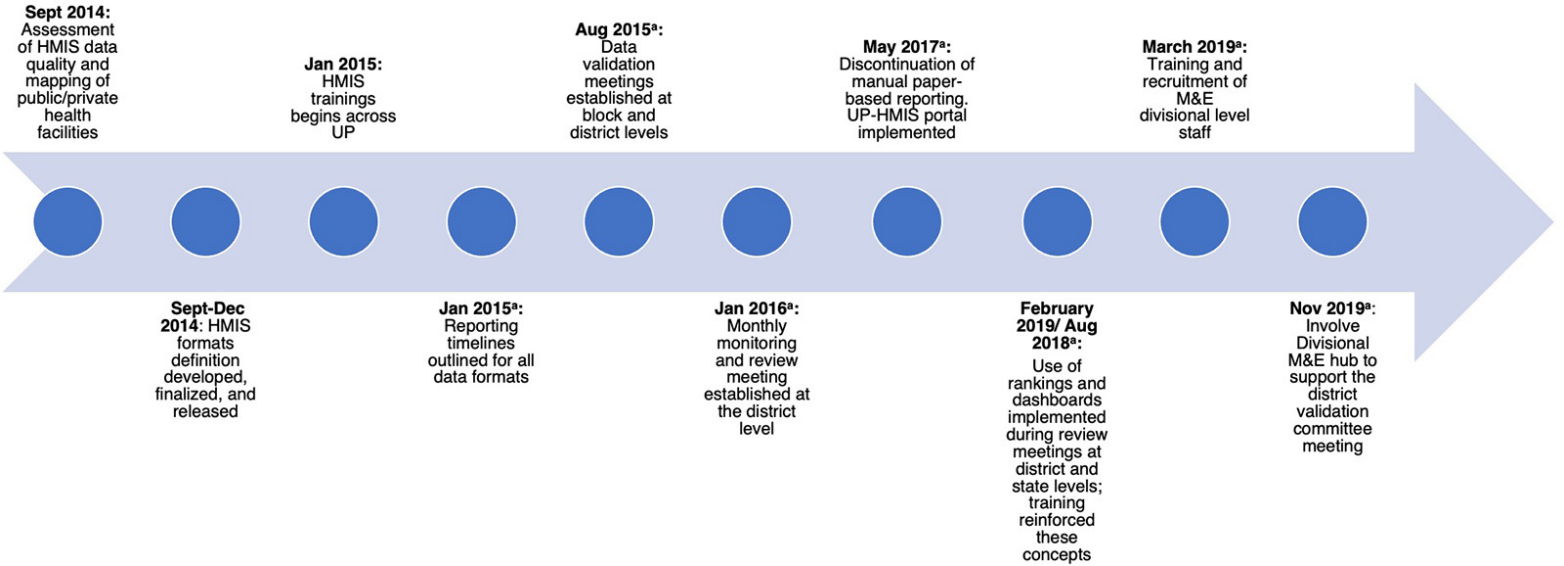
Timeline of the Implementation of Major Activities and Policy Guidelines to Strengthen the Performance of UP-HMIS Abbreviations: GOUP, Government of Uttar Pradesh; HMIS, health management information system; M&E, monitoring and evaluation; UP, Uttar Pradesh; UP-HMIS, Uttar Pradesh Health Management Information System. ^a^Policy guidelines released by the GOUP.

Strong government leadership and an enabling policy environment were critical for the implementation of the new UP-HMIS, as were data quality and review processes to address the organizational determinants of its performance. Government policies, issued as circulars, guidelines, and letters, were important in mobilizing these changes. Figure 2 provides a timeline of major activities and policy guidelines aimed at strengthening UP-HMIS implementation and scale-up.

Specifically, the first set of government policies focused on improving data availability. Government guidelines aimed to promote uniformity of reporting periods across public and private health facilities, increase the use of standardized UP-HMIS formats, and discontinue any manual reporting forms.

Government guidelines aimed to promote uniformity of reporting periods across public and private health facilities, increase the use of standardized UP-HMIS formats, and discontinue any manual reporting forms.

Policy guidelines also targeted increasing reporting from private health facilities. To promote the compliance of HMIS reporting from private medical colleges and private medical facilities at the district level, district offices invited private facilities to participate in a workshop under the chairmanship of the district magistrate and the chief medical officer. This engagement not only sensitized facilities to the guidelines but also created an additional opportunity to update the listings in the HMIS and UP-HMIS as well as the Mother and Child Tracking System, now known as the Reproductive Child Health Portal.

Subsequent government orders focused on institutionalizing processes to enhance data quality and use for decision making at different levels, including at the facility level, where it can influence health care quality. Policy guidelines outlined the composition of data validation committee meetings at the block, district, and state levels, as well as the analysis that should be conducted to assess the absence, completeness, and accuracy of UP-HMIS data. Monitoring guidelines also focused on the implementation of supportive supervision checklists and data audits to enhance data quality across different levels of the health system.

The GOUP released specific guidelines and held trainings to sensitize data-related managers at the block, district, and state levels on how to review, analyze, and monitor the quality of data sources and conduct monthly data validation meetings. In addition, the GOUP issued guidelines to strengthen the use of these validated data. Major policy decisions focused on (1) regularizing the program review meetings, known as executive committee meetings or monthly block medical-officer-in-charge meetings; (2) recommending the review of district and block rankings on the state health dashboard developed using UP-HMIS data; and (3) conducting an action plan based on the analysis of these rankings.

**Figure:**
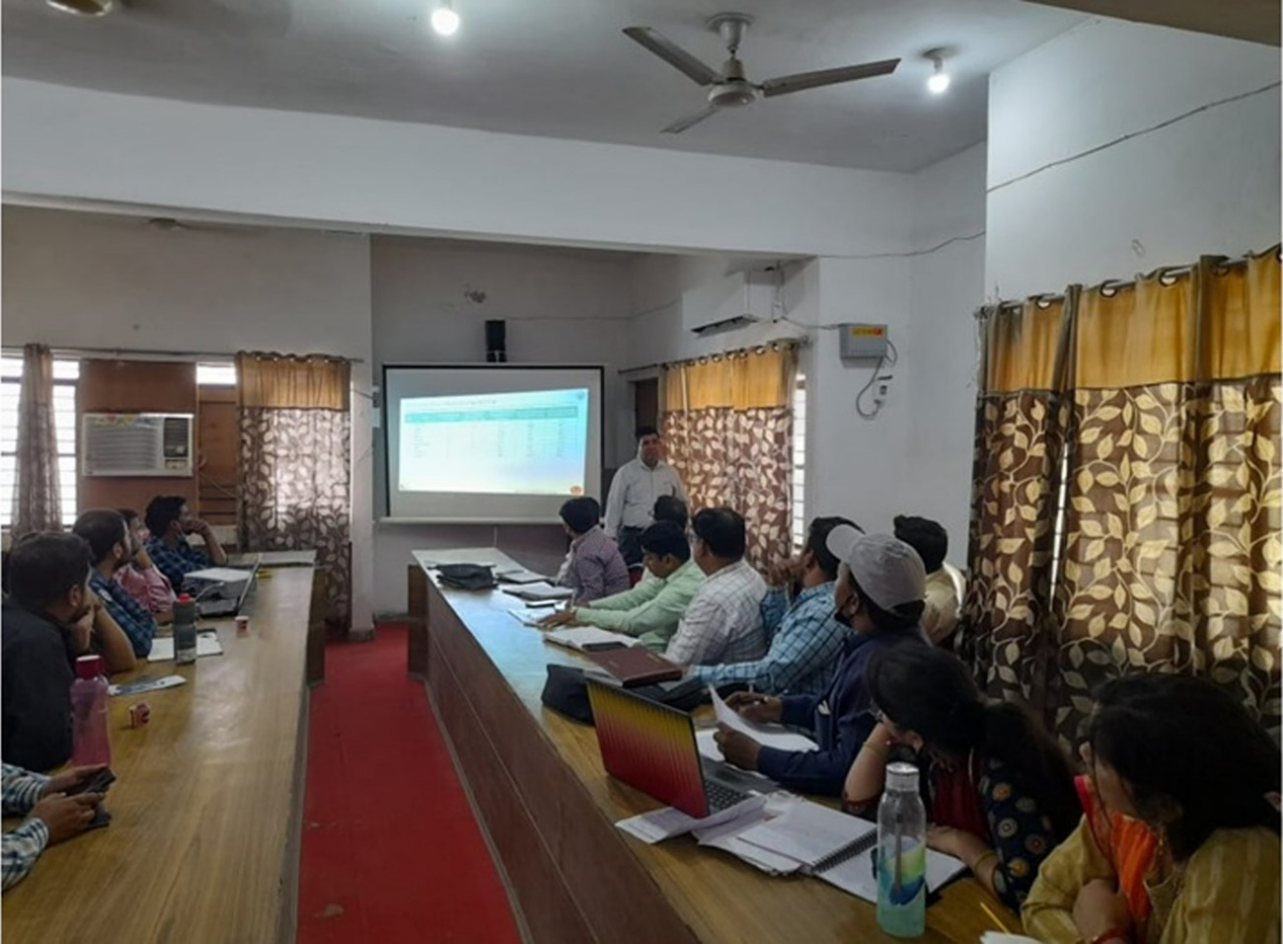
District program manager in Etah district, with support of the monitoring and evaluation specialist, facilitates a district review meeting using the Uttar Pradesh Health Dashboard. © 2022 Shekhar Dutt Sharma, Department of Medical Health and Family Welfare, Uttar Pradesh

#### Providing Technical Assistance to Strengthen District-Level Capacities to Implement the UP-HMIS

UP-TSU’s M&E specialists in the 25 high-priority districts supported the field implementation of the government orders and guidelines for UP-HMIS strengthening activities. Specifically, they facilitated the printing of the revised formats and ensured their availability from the district level to the subcenter levels, which had previously been identified as a barrier. In addition, they supported the planning and implementation of facility-level trainings in those districts.

The UP-TSU and the GOUP also conducted state-wide trainings for subcenter, block, district, division, and state staff involved in data reporting, entry, analysis, and review using the new data formats and portals over a 3-month period in 2017.

Trainings were implemented across all 75 districts using a training-of-trainers model. The UP-TSU staff first trained all district- and block-level staff, who then conducted facility-level trainings for the staff nurses and auxiliary nurse midwives working within their respective jurisdictions. The UP-TSU monitored these facility-level trainings. Overall, these trainings focused on reviewing the key components of the UP-HMIS formats for staff based at the primary health center, community health center, and district hospital levels. Block- and district-level managers, such as the medical officers-in-charge, chief medical officers, program nodal officers, district program managers, and assistant research officers, were additionally trained on how to use the UP-HMIS online portal to review data and generate reports. This training activity also targeted division- and state-level program managers and directors, monitoring and evaluation specialists, and assistant research officers. At least 87% of all targeted participants attended the trainings (Table 3), and for those who missed the trainings, separate trainings were conducted in subsequent months.

**TABLE 3.** Participants Trained on the UP-HMIS, 2017–2018^a^

	**Eligible Participants**	**Total Participants Targeted, No.**	**Training Attendees, No. (%)**
**State**	Program managers, directors, assistant research officers, monitoring & evaluation specialists	73	65 (89)
**Division**	Additional directors	48	48 (93)
**District**	Chief medical officers, chief medical superintendent, assistant chief medical officers, assistant research officers, district program managers, HMIS operator	225	233 (104)
**Block**	Block program managers, block assistant research officers, HMIS operator	2,616	2,431(93)
**Subcenter**	Auxiliary nurse midwives, nurse supervisors	18,028^b^	15,738 (87)
**Total**		20,990	18,515 (88)

Abbreviations: HMIS, health management information system; UP-HMIS, Uttar Pradesh Health Management Information System.

^a^Source: Participant attendance sheet.

^b^Only 1 auxiliary nurse midwife (ANM) per subcenter was targeted for the training (if more than 1 ANM was on staff). For all other positions, everyone was invited to participate in the trainings.

#### Implementing Targeted Initiatives to Strengthen Processes for Data Quality

Following the initial UP-HMIS capacity building and training activities, the GOUP’s efforts focused on strengthening existing data quality processes. First, to establish a platform for monitoring and analyzing the quality of routine data sources on a regular basis, the GOUP implemented monthly data validation committee meetings at the block and district levels and quarterly state-level data quality meetings. These meetings focused on 2 primary objectives: (1) monitoring and rectifying persistent data reporting challenges and (2) ensuring data accuracy by conducting validation checks with source documents.

Following the initial UP-HMIS capacity building and training activities, the GOUP’s efforts focused on strengthening existing data quality processes.

More broadly, to monitor UP-HMIS data quality processes across all the 75 districts in the state, the UP-TSU implemented 2 key assessments: (1) maternal and newborn complications audit to monitor facility-level data quality (implemented in October–November 2017); and (2) the UP-HMIS checklist tool at the facility level (implemented in December 2017–January 2018). A UP-TSU analysis showed data quality improvements (Table 4); subsequently, these activities were integrated into the GOUP’s routine processes.

**TABLE 4.** Improvement in the Data Accuracy of MNCH Data Elements Between Rounds 1 and 2 Observed During Data Audits Conducted by the UP-TSU Across 130 Facilities in 25 High-Priority Districts^a^

**Facility Type**	**Number of MNCH DataElements Audited**	**Number of Facilities Audited for Data Accuracy**	**Data Elements Matched With Source (Round 1,October 2017)**		**Data Elements Matched With Source (Round 2,January 2018)**		**Mean Difference (Round 2–Round 1)**	***P* Value**
			**Mean, %**	**SD**	**Mean, %**	**SD**		
**District hospitals**	98	26	47	32	72	28	25	.007
**Block-level community health center and block primary health care**	97	58	52	36	70	29	17	.001
**Community health center**	97	20	51	37	66	33	15	.178
**Primary health center**	97	17	33	38	69	39	36	.002
**Subcenter**	97	9	53	40	70	37	17	.142
**Total**		130	49	36	70	31	21	.000

Abbreviations: MNCH, maternal, neonatal, and child health; SD, standard deviation; UP-TSU, Uttar Pradesh Technical Support Unit.

a Source: Based on the analysis of Maternal and Newborn Complication Data Audit conducted from Round 1 (October 2017) to Round 2 (January 2018) by the UP-TSU. The 2 rounds of data quality assessment (data audit) for MNCH data elements were conducted by UP-TSU independently. During the audits, selected data elements were matched between reported data and source register data for accuracy.

The implementation of these data quality checks alongside the district data validation committee meetings has enabled data quality to improve month by month. Specifically, blocks and facilities with major data quality issues identified during meetings are prioritized for supervisory visits to identify the sources of these issues. Following the visit, an action plan is designed and shared during the next meeting so that learnings can be shared with other blocks and facilities facing similar challenges.

Currently, UP-TSU M&E specialists remain stationed in the 25 high-priority districts and provide technical support to district-level staff responsible for compiling data. They also review the district’s data quality and support its use to inform programmatic decision making.

#### Implementing Targeted Initiatives to Strengthen Processes for Data Use

Similarly, to strengthen the routine use of these data at block and district levels, the GOUP formally strengthened the existing executive committee platform review meetings (also known as monthly medical-officer-in-charge review meetings), during which district leadership reviews block-level health program performance after the data validation committee meetings. To support this review process, the GOUP (with the support of the UP-TSU) developed an Uttar Pradesh Health Dashboard, which ranks blocks and districts on a key set of priority health and data quality indicators (Supplement 2). These rankings are meant to bring attention to generally low- and high-performing blocks, so that gaps in service delivery (e.g., availability of commodities, drugs, and human resources) can be identified. This facilitates the development of action plans to determine why these gaps exist and how to address them, as well as tracking the decisions made during these meetings to create responsibility and accountability for follow-through.

**FIGURE 3:**
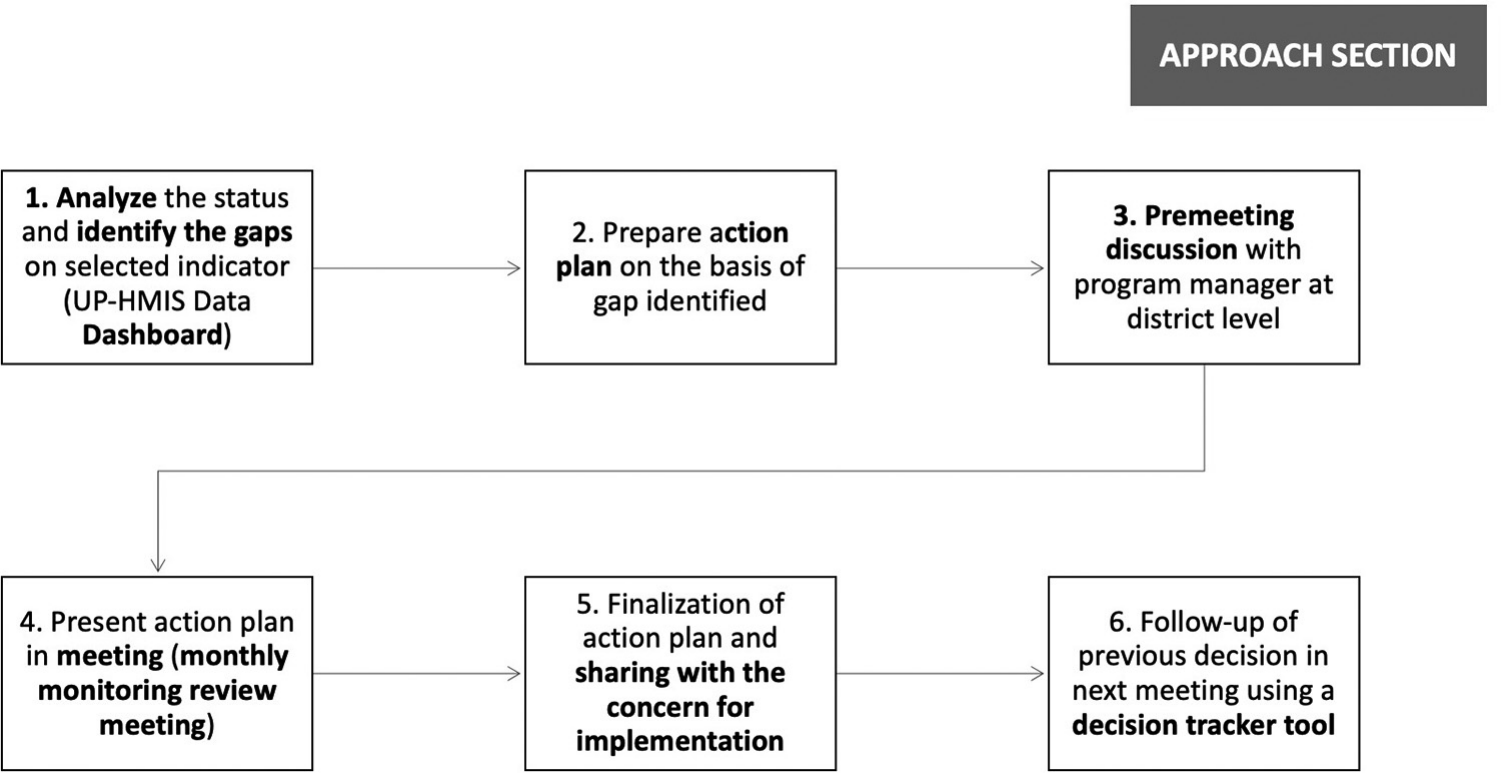
Process of Enhancing the Use of Data for Decision Making During Program Review Meetings at the District Level Abbreviation: UP-HMIS, Uttar Pradesh Health Management Information System.

Figure 3 summarizes the GOUP’s key steps and guidelines for implementing the program review meetings at the district level. To strengthen these data review processes, the UP-TSU has also conducted trainings across high-priority and non-priority districts in Uttar Pradesh. These trainings have focused on using data for decision making, including how to download, analyze, and interpret the data for a set of indicators according to program areas.

## UP-HMIS ACHIEVEMENTS AND RELATED CAPACITY-STRENGTHENING INITIATIVES

### Improvements in Data Completeness and Quality

**FIGURE 4:**
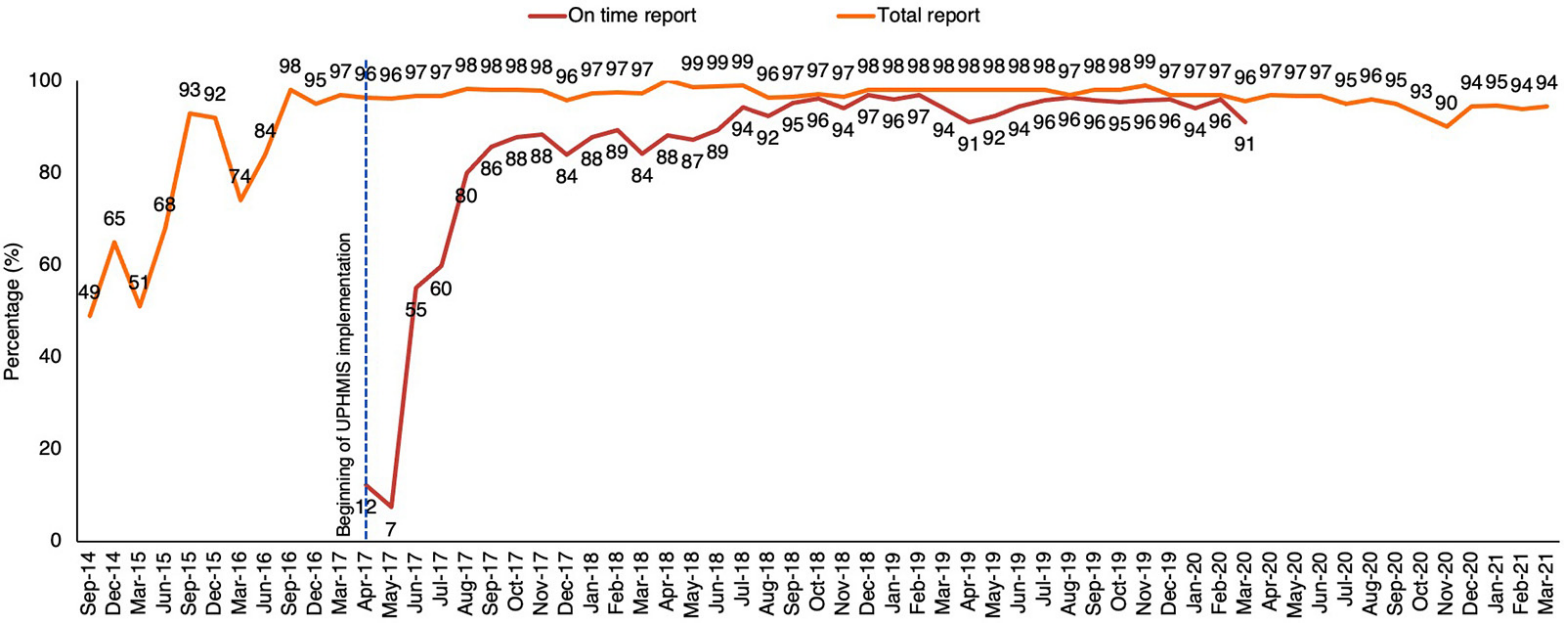
Percentage of All Facilities in Uttar Pradesh Reporting Data^a,b^ in the UP-HMIS and HMIS Portals, September 2014–March 2021 Abbreviations: HMIS, health management information system; UP-HMIS, Uttar Pradesh Health Management Information System. ^a^On-time reporting defined as “% of facilities which have uploaded data on portal as on 30th of the month.” This indicator was tracked for HMIS portal (before April 2017) and UP-HMIS portal (after April 2017) from across the 75 districts in the state. ^b^Total reporting defined as “% of facilities which have ever reported for the specified month on the portal.” Prior to 2017, the “total report” refers to total reports of HMIS formats. Following 2017, the “total report” refers to the UP-HMIS format, which include both HMIS and new UP-HMIS data elements.

**FIGURE 5:**
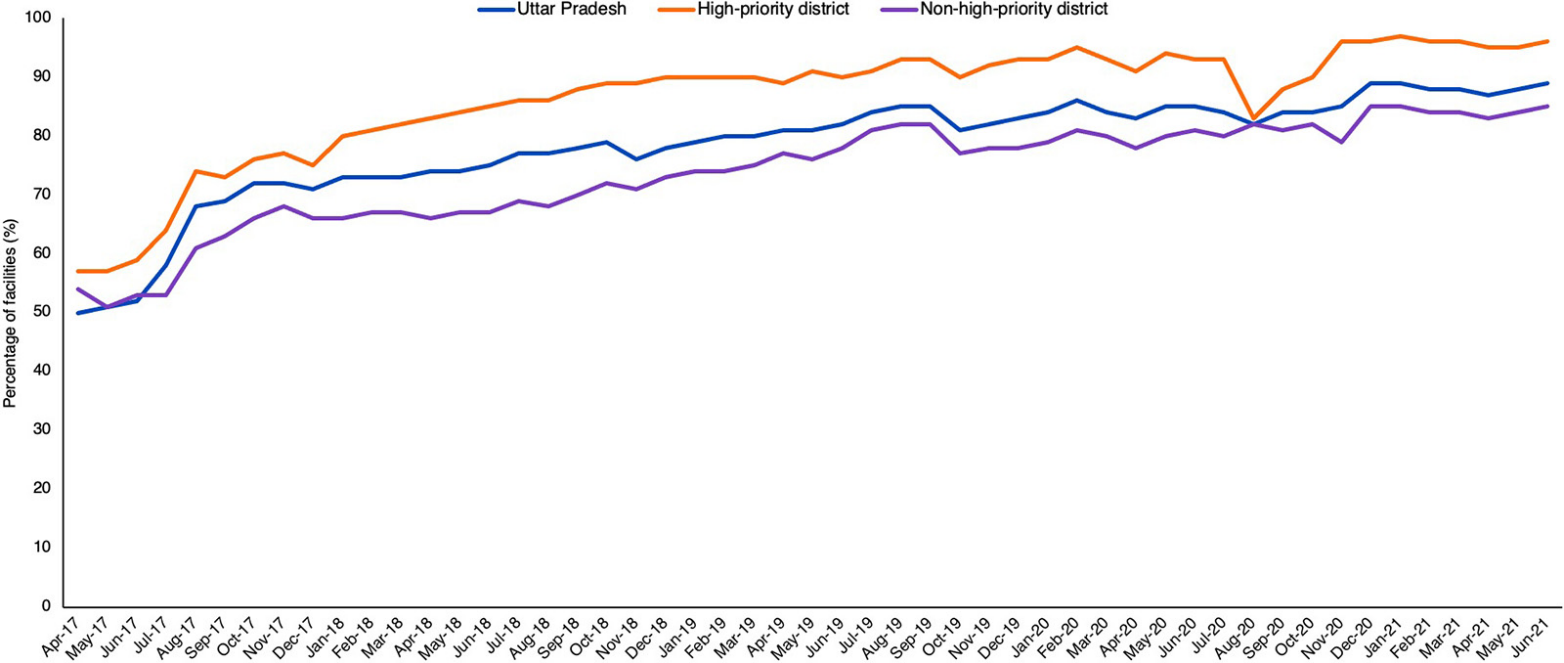
Percentage of Total Facilities With Greater Than 80% Non-Blank Data Element (Data Completeness)^a,b^ Abbreviation: UP-HMIS, Uttar Pradesh Health Management Information System. ^a^Source: UP-HMIS data quality analysis. ^b^Completeness data after March 2020 is not comparable because HMIS data began being captured using a mobile/tablet application at the source in phased manner across districts (as opposed to being entered on paper and then reentered on the web-based UP-HMIS portal).

Since the creation of UP-HMIS, more facilities report data on time, more completely, and with fewer errors. Data entered into the UP-HMIS are used to generate a spreadsheet that is imported to the national HMIS portal, minimizing discrepancies between UP-HMIS and HMIS data. There have been improvements in other data quality metrics as well. For example, on-time UP-HMIS reporting from health facilities has increased from 12% in 2017 to above 90% by 2020 (Figure 4). Similarly, there has been a steady increase in the total reporting (defined as facilities that have ever reported for specific months) to the portal, which has increased from 49% in 2014 (in HMIS) and has consistently remained above 90% since the implementation of UP-HMIS (Figure 5).^15^

Since the creation of UP-HMIS, more facilities report data on time, more completely, and with fewer errors.

Several data quality comparisons examined changes over time and across districts, using data collected during supervision visits and data audits: All showed improvements in data accuracy. A data audit assessment (Supplement 3) showed that the accuracy of data elements reported in UP-HMIS relating to delivery, complication management and referral, and pregnancy outcomes significantly improved from 49% in October 2017 to 70% in January 2018 (Table 4). Similarly, differences between the proportion of data elements matched with the source document also decreased substantially between October 2017 and January 2018 in the 25 high-priority districts (Table 4). Third, the comparison of 24 critical data elements related to reproductive, maternal, and child health services (as reported in 2 rounds of supportive supervision in 8 aspirational districts of Uttar Pradesh) indicated that data accuracy improved from 62% in July 2018 to 74% in February 2020.^16^

### Improvements in Data Use

There is evidence that UP-HMIS is being used by district officials across the state. In April–May 2019, the UP Health Dashboard user log and an assessment conducted by UP-TSU found that 23 of 25 high-priority districts were also actively conducting gap analysis and preparing action plans based on dashboard and UP-HMIS data, as well as conducting review meetings on this basis (data not shown). Similarly, during district-level executive committee review meetings, the assessment revealed that district leadership (namely, the district magistrate and chief medical officer) frequently referred to the UP Health Dashboard to make decisions. The UP-TSU found that between November 2017 and September 2019, the 25 high-priority districts made 631 decisions based on data (Supplement 4).

## REFLECTIONS ON ACHIEVEMENTS, CHALLENGES, AND THE WAY FORWARD

Overall, we hope this article offers insights into the steps taken to identify, develop, and incorporate relevant data by designing a state’s HMIS, and developing and implementing supportive processes to improve the quality and use of those data in decision making.

One of the GOUP’s strategic goals has been to increase the availability, quality, and use of routine health data for improving program implementation. To make progress toward this goal, the GOUP, in collaboration with the UP-TSU, developed UP-HMIS, which addressed the national HMIS’ data gaps by ensuring that relevant data were also being collected to support programmatic decision making at the lower levels of the health system. While the UP-HMIS provided district- and state-level decision makers with the data required to manage and track health program performance, the GOUP also rolled out interventions designed to address organizational factors and improve processes for data quality and use to ensure the benefits of the UP-HMIS were fully realized.

Government orders, guidelines, and policies institutionalized data quality and review meetings at the district and state levels. The UP-TSU provided technical assistance to conduct the district-level meetings and more broadly attempted to lay the groundwork for an organizational culture that values quality data use in decision making. For example, the GOUP (in collaboration with the UP-TSU) conducted training activities across all levels of the health system to ensure sufficient understanding and use of these data in decision making by health workers; block-, district-, and state-level program managers; and data staff.

Together, these initiatives contributed to improvements in data availability, quality, and use, and led to a positive shift in how data quality is checked and reviewed at the block and district levels in Uttar Pradesh.

However, several data quality challenges persist. For example, human resource shortages and gaps in technical skills have affected the data quality and use meetings at the block and district levels.^17^ Working within a very hierarchical organizational system where the focus is on achieving district rankings has also resulted in data quality processes being overlooked or even ignored unless there is sufficient demand from leadership.^17^ Addressing these challenges will require institutionalizing the UP-HMIS and related data quality and use processes. First, periodic review of the UP-HMIS formats and the rationalization of the data elements will be important to ensure that only relevant and necessary information is collected, thereby reducing the data reporting burden. Second, regular data quality audits need to be conducted to track the quality of the government data systems, particularly private-sector facilities, which are still underreporting into the UP-HMIS. Third, these data quality processes can be strengthened by ensuring that presiding district- and block-level staff have sufficient time and resources for supportive supervision activities.^18^ To support these processes, the GOUP has also established a data monitoring hub at the division level (the administrative tier between the district and state levels) to bring greater attention to issues of data quality. The division monitoring hub supports district program managers by analyzing data but also conducts supportive supervision activities at the district and block levels and implements periodic data quality audits to ensure accuracy.

Addressing persisting data quality challenges will require institutionalizing the UP-HMIS and related data quality and use processes.

With respect to data use, the UP Health Dashboard has been critical in incentivizing blocks and districts to pay closer attention to the performance of the 14 ranking health program indicators during data review meetings. In several districts, action plans are reportedly being developed to address gaps that may be affecting indicator performance. The GOUP and UP-TSU will also expand the data review meetings and processes to lower levels of the health system, including health workers who will be trained to use and analyze the data as a job aid for planning.

Fully benefiting from the UP Health Dashboard and these data review initiatives will require developing a culture of data use culture across the state, which continues to remain GOUP’s long-term vision. Additional investments will be required to promote behavioral change among health managers in the public health system but also among private-sector providers who report data to the UP-HMIS.^18^ Several trainings have also focused on improving data use for decision making to increase the capacity of government-funded staff to conduct data analysis; the eventual goal is to ensure data use across all districts, with limited need for UP-TSU’s technical assistance.

Institutionalizing processes for data quality and use and building an organizational culture that values good quality data for decision making will happen over a considerable period. The GOUP views the development of the UP-HMIS as a first step toward centralizing the state’s health data.

The GOUP has already started addressing some of the technical gaps in data collection, quality, and use by reducing paper-based reporting and attempting to integrate multiple data collection platforms. For example, with the introduction of new systems like data entry at source (via a mobile-based subcenter application) and an e-HMIS (e-Hospital Management Information System), which will allow tracking of all services received by patients in the facility, the quality and use of data are expected to improve.

Greater investments are required to achieve this vision and to improve the quality of these data; the expectation is that data use will help improve data availability and quality over time.^19^ These investments will be important for the GOUP to achieve its long-term vision of developing an integrated information communication technology architecture to allow the flow of health data across different data portals into a central dashboard repository that visually presents key program monitoring data to support management and decision making at various levels in the health system (unpublished data).

## Supplementary Material

GHSP-D-21-00632-supplement.pdf

## Notes

**Peer Reviewed**

**First published online**: August 23, 2022.
