# Supplementary material for: Optimizing the Health Management Information System in Uttar Pradesh, India: Implementation Insights and Key Learnings
Source: Glob Health Sci Pract. 2022 Aug 30;10(4):e2100632. doi: 10.9745/GHSP-D-21-00632 (PMC9426977; doi:10.9745/GHSP-D-21-00632)
Supplement: GHSP-D-21-00632-supplement.pdf [file GHSP-D-21-00632-supplement.pdf]

Supplement to: Meghani A, Tripathi AB, Bilal H, et al. Optimizing the health management information system in Uttar Pradesh, India: implementation insights and key learnings. *Glob Health Sci Pract*. 2022;10(4):e2100632. https://doi.org/10.9745/GHSP-D-21-00632

SUPPLEMENTS

Supplement 1. Increase in the number of public and private health facility included in the HMIS/UP-HMIS platform, expected to submit monthly reports, following the UP-HMIS systems strengthening activities conducted by the UP-TSU in 2013-2014

Facility type	2014-15 (HMIS)	2015-16 (HMIS)	2016-17 (HMIS)	2017-18 (UP-HMIS)	2018-19 (UP-HMIS)
**Sub center**	20,487	20,769	20,784	20,763	20,792
**Primary Health Center**	2,069	3,081	3,149	3,257	3,406
**Community Health Center** [a]	636	625	638	646	677
**Sub District Hospital**[b]	6	6	5	0	0
**District Hospital**	147	158	162	173	174
**Private**[c,d]	75	514	2,357	2,193	2,514
**Total**	23,420	25,153	27,095	27,032	27,563

[a] Decline in the number of community health centers 2015-16 was attributed to mapping of private health facilities as subcenters which was corrected in subsequent years. Increases are attributed to the opening of new community health centers
[b] Sub-district hospitals do not exist in Uttar Pradesh, however HMIS which is a Government of India reporting form lists this as a category. This misclassification was rectified in the reporting forms in 2017, and therefore shows 0 sub-district hospitals since this entity does not exist in UP
[c] Private health facilities include allopathic service providers, who are registered with district health offices and provide reproductive child health services.
[d] Changes in the number of private facilities reflect new data on private health facilities collated during the annual renewal of the facilities by district chief medical officer's office.

Source: UP-TSU analyses

Supplement to: Meghani A, Tripathi AB, Bilal H, et al. Optimizing the health management information system in Uttar Pradesh, India: implementation insights and key learnings. *Glob Health Sci Pract*. 2022;10(4):e2100632. https://doi.org/10.9745/GHSP-D-21-00632

Supplement 2. Indicators included in the district rankings presented in the UP Health Dashboard in January 2019

#	Domain	Indicator	Type	Data Source
1	Ante Natal	% of pregnant women received 4 or more antenatal care (ANC) and tested for Hb against estimated pregnant women (PW)	*Coverage*	UP-HMIS[1]
1A		*% of pregnant women received 4 or more ANC against estimated PW*	*Coverage*	*UP-HMIS*
1B		*% of pregnant women tested for Hb for 4 or more times against estimated PW*	*Quality*	*UP-HMIS*
2	Delivery care	% of pregnant women delivered in institution against estimated delivery	Coverage	UP-HMIS
3		% of C-section delivery against reported delivery (70% weightage to CHC and 30% to DH)	Quality	UP-HMIS
4		Still birth ratio	Quality	UP-HMIS
5	Postnatal care	% of women receiving postpartum check within 48 hour of home delivery against reported delivery (Home +institutional)	Coverage	UP-HMIS
6	Immunization	Ratio of Pentavalent 3 to BCG	Quality	UP-HMIS
7		% of children received full immunization	Coverage	UP-HMIS
8	Family Planning	% of eligible couple accepted limiting method	Coverage	UP-HMIS
9		% of eligible couple accepted spacing method	Coverage	UP-HMIS
10	Communicable Disease	Total case notification rate of TB against expected TB cases	Quality	RNTCP MIS[2]
11		% of PW screened for HIV against estimated pregnancy	Coverage	UP-HMIS
12	Fund utilization	Per ASHA expenditure of ASHA incentive fund	Coverage	NHM FMR[3]
13	Data quality: Completeness	% of units reported non blank value (including zero) for the identified indicators of ranking	Data quality	UP-HMIS
14	Data quality: Consistency	% of units reported outlier for the identified indicators of ranking	Data quality	UP-HMIS

[1]Uttar Pradesh- Health Management Information Systems; [2]Revised National TB Control Programme Management Information System is a case-based web-based TB surveillance system also known as "NIKSHAY" ; [3]National Health Mission Financial Management Reports.

Note: Data are imported from identified portals for indicator 7, 10 and 12 into the UP-HMIS system to generate district rankings

Source: UP-TSU analyses

Supplement to: Meghani A, Tripathi AB, Bilal H, et al. Optimizing the health management information system in Uttar Pradesh, India: implementation insights and key learnings. *Glob Health Sci Pract*. 2022;10(4):e2100632. https://doi.org/10.9745/GHSP-D-21-00632

Supplement 3. Maternal and Newborn Complication Data Audit tool

MATERNAL AND NEWBORN COMPLICATION DATA AUDIT

A. IDENTIFICATION DETAILS

District____________________________ District Code

Block____________________________ Block Code

Name of Facility__________________________ Facility Code

Type of Facility_______________________(DH/CHC-FRU/CHC/PHC/APHC/SC/OTHER)

Name of Data Auditor ____

Designation____

Name of Facility In-Charge ______________________________________

Mobile Number of Facility In charge ________________________________

Name of Nurse Mentor____Mobile Number__________________________

Date of Visit___________________________Day _______________________________

**B.**	**General Information**	RESPONSE
#Q	Information- To be collected from records and Observation	
B.1	Average monthly delivery of last 6 month (Source- Register)	
B.2	Number of Staff Nurse Posted in Labor Room	
B.3	Number of ANM Posted in Labor Room	
B.4	Number of Labor Table Available in Labor room	
B.5	Functional Radiant Warmer Available	(Yes/No)

Supplement to: Meghani A, Tripathi AB, Bilal H, et al. Optimizing the health management information system in Uttar Pradesh, India: implementation insights and key learnings. *Glob Health Sci Pract*. 2022;10(4):e2100632. https://doi.org/10.9745/GHSP-D-21-00632

C. Monthly Facility Report – Data Audit						
**#**	**Data Element**	**Value as per HMIS/ UPHMIS**	**Source Document Available (Yes/No)**	**Value as Per Source Document**	**Name of Source Document**	**If not matched, data corrected on portal (Y/N/M)**
1	**Arrival and Admission**					
1.1	Pregnant woman arrived in the facility -In Labor					
1.2	Pregnant woman arrived in the facility –Not in Labor					
1.3	Pregnant woman admitted in the facility- In labor					
1.4	Pregnant woman admitted in the facility - Not in labor					
1.5	Case sheet filled for deliveries conducted at the facility					
2	**Outcome among arrival PW**					
2.1	Referred to higher facility					
2.2	Sent home healthy					
2.3	Deaths at facility					
3	**Delivery Detail**					
3.1	Total Delivery conducted					
3.2	Total C-Section deliveries performed					
3.3	Total live birth					
3.4	Still birth : Fresh					
3.5	Still birth : Macerated					
3.6	Total Still Birth					
4	**Outcome among New born**					
4.1	Referred to higher facility- Newborn					
4.2	Sent home healthy - Newborn					
4.3	Deaths at facility - Newborn					
5	**Maternal Complication**					
5.1.1	Prolonged labor: Sent home healthy after discharge					
5.1.2	Prolonged labor: LAMA					
5.1.3	Prolonged labor: Died at facility					
5.1.4	Prolonged labor: Referred and survived					
5.1.5	Prolonged labor: Referred and Died					
5.1.6	Prolonged labor: Referred and Status unknown					

Supplement to: Meghani A, Tripathi AB, Bilal H, et al. Optimizing the health management information system in Uttar Pradesh, India: implementation insights and key learnings. *Glob Health Sci Pract*. 2022;10(4):e2100632. https://doi.org/10.9745/GHSP-D-21-00632

#	Data Element	Value as per HMIS/ UPHMIS	Source Document Available(Yes/ No)	Value as Per Source Document	Name of Source Document	If not matched, data corrected on portal (Y/N/M)
5.2.1	Obstructed labor: Sent home healthy after discharge					
5.2.2	Obstructed labor: LAMA					
5.2.3	Obstructed labor: Died at facility					
5.2.4	Obstructed labor: Referred and survived					
5.2.5	Obstructed labor:Referred and Died					
5.2.6	Obstructed labor: Referred and Status unknown					
5.3.1	Pregnancy induced hypertension: Sent home healthy after discharge					
5.3.2	Pregnancy induced hypertension: LAMA					
5.3.3	Pregnancy induced hypertension: Died at facility					
5.3.4	Pregnancy induced hypertension: Referred and survived					
5.3.5	Pregnancy induced hypertension:Referred and Died					
5.3.6	Pregnancy induced hypertension:Referred and Status unknown					
5.4.1	Mild pre-Eclampsia: Sent home healthy after discharge					
5.4.2	Mild/Severe pre-Eclampsia: LAMA					
5.4.3	Mild/Severe pre-Eclampsia: Died at facility					
5.4.4	Mild/Severe pre-Eclampsia: Referred and survived					
5.4.5	Mild/Severe pre-Eclampsia: Referred and Died					
5.4.6	Mild/Severe pre-Eclampsia: Referred and Status unknown					
5.5.1	Eclampsia: Sent home healthy after discharge					
5.5.2	Eclampsia: LAMA					
5.5.3	Eclampsia: Died at facility					
5.5.4	Eclampsia: Referred and survived					
5.5.5	Eclampsia: Referred and Died					
5.5.6	Eclampsia: Referred and Status unknown					
5.6.1	Antepartum hemorrhage (APH):Sent home healthy after discharge					
5.6.2	Antepartum hemorrhage (APH):LAMA					

Supplement to: Meghani A, Tripathi AB, Bilal H, et al. Optimizing the health management information system in Uttar Pradesh, India: implementation insights and key learnings. *Glob Health Sci Pract*. 2022;10(4):e2100632. https://doi.org/10.9745/GHSP-D-21-00632

5.6.3	Antepartum hemorrhage (APH):Died at facility					
5.6.4	Antepartum hemorrhage (APH):Referred and survived					
5.6.5	Antepartum hemorrhage (APH):Referred and Died					
5.6.6	Antepartum hemorrhage (APH):Referred andStatus unknown					
#	**Data Element**	**Value as per HMIS/ UPHMIS**	**Source Document Available(Yes/ No)**	**Value as Per Source Document**	**Name of Source Document**	**If not matched, data corrected on portal (Y/N/M)**
5.7.1	Infection / sepsis: Sent home healthy afterdischarge					
5.7.2	Infection / sepsis: LAMA					
5.7.3	Infection / sepsis: Died at facility					
5.7.4	Infection / sepsis: Referred and survived					
5.7.5	Infection / sepsis: Referred and Died					
5.7.6	Infection / sepsis: Referred and Status unknown					
5.8.1	PPH: Sent home healthy after discharge					
5.8.2	PPH: LAMA					
5.8.3	PPH: Died at facility					
5.8.4	PPH: Referred and survived					
5.8.5	PPH: Referred and Died					
5.8.6	PPH: Referred and Status unknown					
5.9.1	Anemia: Sent home healthy after discharge					
5.9.2	Anemia: LAMA					
5.9.3	Anemia: Died at facility					
5.9.4	Anemia: Referred and survived					
5.9.5	Anemia: Referred and Died					
5.9.6	Anemia: Referred and Status unknown					

Supplement to: Meghani A, Tripathi AB, Bilal H, et al. Optimizing the health management information system in Uttar Pradesh, India: implementation insights and key learnings. *Glob Health Sci Pract*. 2022;10(4):e2100632. https://doi.org/10.9745/GHSP-D-21-00632

#	Data Element	Value as per HMIS/ UPHMIS	Source Document Available	Value as Per Source Document	Name of Source Document	If not matched, data corrected on portal (Y/N/M)
6	**Newborn Complication**					
6.1.1	Preterm birth (<37 weeks gestation): Sent homehealthy after discharge					
6.1.2	Preterm birth (<37 weeks gestation): LAMA					
6.1.3	Preterm birth (<37 weeks gestation): Died at facility					
6.1.4	Preterm birth (<37 weeks gestation): Referred and survived					
6.1.5	Preterm birth (<37 weeks gestation): Referred and Died					
6.1.6	Preterm birth (<37 weeks gestation): Referred and Status unknown					
6.2.1	Low birth weight (<2500 grams): Sent home healthy after discharge					
6.2.2	Low birth weight (<2500 grams): LAMA					
6.2.3	Low birth weight (<2500 grams): Died at facility					
6.2.4	Low birth weight (<2500 grams): Referred and survived					
6.2.5	Low birth weight (<2500 grams): Referred and Died					
6.2.6	Low birth weight (<2500 grams): Referred and Status unknown					
6.3.1	Asphyxia: Sent home healthy after discharge					
6.3.2	Asphyxia: LAMA					
6.3.3	Asphyxia: Died at facility					
6.3.4	Asphyxia: Referred and survived					
6.3.5	Asphyxia: Referred and Died					
6.3.6	Asphyxia: Referred and Status unknown					
6.4.1	Birth anomalies: Sent home healthy after discharge					
6.4.2	Birth anomalies: LAMA					
6.4.3	Birth anomalies: Died at facility					
6.4.4	Birth anomalies: Referred and survived					
6.4.5	Birth anomalies: Referred and Died					
6.4.6	Birth anomalies: Referred and Status unknown					
7	**Blood bank services**					
7.1	Functional blood bank unit (Applicable for DH)					
7.2	Functional blood storage unit					

7.3	Number of complicated pregnancy treated withblood transfusion					

Supplement to: Meghani A, Tripathi AB, Bilal H, et al. Optimizing the health management information system in Uttar Pradesh, India: implementation insights and key learnings. *Glob Health Sci Pract*. 2022;10(4):e2100632. https://doi.org/10.9745/GHSP-D-21-00632

Action Plan

S.NO	Section	Gap Identified	Person Responsible	Timeline	Status
1					
2					
3					
4					
5					
6					
7					
8					
9					
10					
11					
12					
13					
14					
15					
16					
17					
18					
19					
20					

Supplement to: Meghani A, Tripathi AB, Bilal H, et al. Optimizing the health management information system in Uttar Pradesh, India: implementation insights and key learnings. *Glob Health Sci Pract*. 2022;10(4):e2100632. https://doi.org/10.9745/GHSP-D-21-00632

Supplement 4. Domain wise decisions taken by District Magistrates and Chief Medical Officers during Program Review meetings in 25 high priority districts from November 2017 to September 2019

Domain	Cumulative number of decisions taken	Percentage
Antenatal care	175	28%
Delivery care	116	18%
Family planning	78	12%
Child health	71	11%
Postnatal and newborn care	61	10%
Other	130	21%
Total	631	100%

Source: UP-TSU analyses based on decision-tracker data collected from 25 HPDs
